# Cross sectional study of the clinical characteristics of French primary care patients with COVID-19

**DOI:** 10.1038/s41598-021-91685-3

**Published:** 2021-06-14

**Authors:** Paul Sebo, Benoit Tudrej, Julie Lourdaux, Clara Cuzin, Martin Floquet, Dagmar M. Haller, Hubert Maisonneuve

**Affiliations:** 1 Primary Care Unit, Faculty of Medicine, University of Geneva, Geneva, Switzerland; 2 University College of General Medicine, University Claude Bernard Lyon 1, Lyon, France; 3 Mermoz Primary Health Centre, Lyon, France; 4 Cerballiance Rhône-Alpes Laboratory, Lyon, France

**Keywords:** Diseases, Health care, Medical research, Signs and symptoms

## Abstract

The early identification of patients suffering from SARS-CoV-2 infection in primary care is of outmost importance in the current pandemic. The objective of this study was to describe the clinical characteristics of primary care patients who tested positive for SARS-CoV-2. We conducted a cross-sectional study between March 24 and May 7, 2020, involving consecutive patients undergoing RT-PCR testing in two community-based laboratories in Lyon (France) for a suspicion of COVID-19. We examined the association between symptoms and a positive test using univariable and multivariable logistic regression, adjusted for clustering within laboratories, and calculated the diagnostic performance of these symptoms. Of the 1561 patients tested, 1543 patients (99%) agreed to participate. Among them, 253 were positive for SARS-CoV-2 (16%). The three most frequently reported ‘ear-nose-throat’ and non-‘ear-nose-throat’ symptoms in patients who tested positive were dry throat (42%), loss of smell (36%) and loss of taste (31%), respectively fever (58%), cough (52%) and headache (45%). In multivariable analyses, loss of taste (OR 3.8 [95% CI 3.3–4.4], p-value < 0.001), loss of smell (OR 3.0 [95% CI 1.9–4.8], p < 0.001), muscle pain (OR 1.6 [95% CI 1.2–2.0], p = 0.001) and dry nose (OR 1.3 [95% CI 1.1–1.6], p = 0.01) were significantly associated with a positive result. In contrast, sore throat (OR 0.6 [95% CI 0.4–0.8], p = 0.003), stuffy nose (OR 0.6 [95% CI 0.6–0.7], p < 0.001), diarrhea (OR 0.6 [95% CI 0.5–0.6], p < 0.001) and dyspnea (OR 0.5 [95% CI 0.3–0.7], p < 0.001) were inversely associated with a positive test. The combination of loss of taste or smell had the highest diagnostic performance (OR 6.7 [95% CI 5.9–7.5], sensitivity 44.7% [95% CI 38.4–51.0], specificity 90.8% [95% CI 89.1–92.3]). No other combination of symptoms had a higher performance. Our data could contribute to the triage and early identification of new clusters of cases.

## Introduction

After its emergence in China in December 2019^1,2^, severe acute respiratory syndrome coronavirus-2 (SARS-CoV-2) hit Europe, which quickly became the epicenter of the epidemic in the spring of 2020. On 11 March 2020, WHO declared the outbreak of coronavirus disease 2019 (COVID-19) a pandemic^3^, and in all countries, primary care and emergency services have had to face the difficult diagnostic challenge defined by this new disease^4^. In France, after the first three cases identified on 24 January 2020 in travelers returning from China^5^, the epidemic spread rapidly throughout the country, and put strong pressure on health systems, particularly hospitals. With more than 100,000 deaths recorded at the end of April 2021, France is among the most bereaved countries in the world^6^.

A large number of publications, some of them relatively recent, allowed us to better characterize the clinical pictures of COVID-19 patients. Yet, most of these studies focused either on the general population^7–9^ or on inpatients^10–13^ or patients presenting to the hospital or outpatient clinic^14–17^. To our knowledge, only a few studies have so far been conducted in primary care, including findings from our group, focusing on smell and taste disorders^18–20^. Several symptoms appear specific to the infection, some of them being relatively frequent (e.g. smell and taste disorders)^19,21,22^, others much rarer (such as frostbite)^23^. It is of utmost importance to gain a better understanding of the specific symptoms of SARS-CoV-2 infection in primary care settings in order to help general practitioners (GPs) triage patients and anticipate medical follow-up.

We aimed to describe the clinical characteristics of primary care patients testing positive for SARS-CoV-2. To do this, we conducted a cross-sectional study in two community-based laboratories located in Lyon (France), equipped to receive primary care patients suspected of being infected with SARS-CoV-2, and compared symptoms of patients with positive and negative RT-PCR tests.

## Methods

### Study site and study population

This cross-sectional study was conducted between March 24 and May 7, 2020 in two community-based laboratories in Greater Lyon (a French city of almost 3 million inhabitants), to which GPs refer patients with suspected COVID-19 for a nasopharyngeal smear (RT-PCR). Following the screening policy in France, only symptomatic patients were referred for testing (after a consultation with their GP). The study population consisted of both these patients and healthcare professionals who generally went directly to the laboratory without prior consultation with a GP. We already published preliminary data from this study, focusing on smell and taste disorders^19^. Here we extend the analysis to the entire sample of patients and the entire range of symptoms they present.

### Data collection

Prior to being tested, patients and healthcare professionals were interviewed by the laboratory nurses using a pre-formatted questionnaire about their gender, age and medical conditions. They were also asked to report their symptoms (by answering yes or no) using a pre-determined list based on previous literature and expert opinion^10,24^. This list was separated into ‘ear-nose-throat’ (ENT) symptoms (i.e., dry throat, dry nose, sore throat, stuffy nose (nasal congestion), loss of taste, loss of smell) and other symptoms (i.e., fever, fatigue, headache, muscle pain, chest pain, palpitations, cough, dyspnea, diarrhea, nausea). There were no exclusion criteria for this study. All patients were eligible for the study, regardless of their age. For the sake of simplicity, all participants in the study are referred to as "patients," whether they are patients per se or healthcare professionals.

### Diagnosis of COVID-19

Diagnosis of COVID-19 was based on the results of real-time RT-PCR (RT-qPCR), using respiratory samples obtained by nasopharyngeal swab. Two diagnostic kits were used in the study: Allplex 2019-nCoV Assay kit (SEEGENE) for the detection of RdRP and N genes specific for SARS-CoV-2 and E gene for all *Sarbecoviruses* including SARS-CoV-2, and Cobas SARS-CoV-2 Assay kit (ROCHE) for the detection of ORFlab gene specific for SARS-CoV-2 and E gene for all *Sarbecoviruses* including SARS-CoV-2^25^. Serologies were not done systematically since these tests were not widely available at the time the data were collected. However, data about serological tests undertaken at a later stage could be retrieved by the lab and linked to the data collected in the study, using the anonymous patient number.

### Confidentiality and ethical approval

All methods were carried out in accordance with relevant guidelines and regulations. Before responding to the questionnaire, all patients (or parents for patients who were minors) provided consent for their anonymous data to be used for research purposes. The study was approved by the Ethics Committee of the *Collège National des Généralistes Enseignants* (number 200423163) and the French CNIL (data protection authority).

### Statistical analyses and sample size

We used frequency tables to summarize sociodemographic characteristics, except for age (median and interquartile range). We then carried out univariate logistic regression adjusted for clustering within the laboratories (using the Stata *vce cluster* command, which uses a robust variance estimator) to examine the association between patient-reported symptoms and SARS-CoV-2 test positivity. After discussion within the research team, in order to focus on common clinical characteristics of COVID-19, we planned to only examine symptoms frequently reported by patients, i.e. reported by at least 5% of patients. Nausea and palpitations were not included in the analysis (reported by less than 1.5% of patients). Using multivariable logistic regression, we adjusted the data for gender, age group, patient population (patient referred by GP or health care provider), RT-PCR date (March, April or May 2020) and all other symptoms frequently reported by patients. Then, we calculated the diagnostic performance (sensitivity, specificity, AUC (Area Under the ROC or Receiver Operating Characteristic Curve), positive and negative predictive values) for all patient-reported symptoms.

We also conducted sensitivity analyses for missing data. We repeated the multivariable analysis after removing the dichotomous variable “patient population” (i.e., patients referred by GP or health care provider) because it had a relatively large amount of missing data. By doing so, the number of missing data was greatly reduced (from 349 to 94, corresponding to only 6.1% missing data).

We calculated the sample size required for our study using the formula for proportions estimated with a given precision. We anticipated symptom prevalence between 10 and 50%, and we wanted to be able to provide a 95% confidence interval width of about 0.05 for the estimate. The minimum required sample size ranged from 553 (for symptoms with 10% prevalence) to 1537 (for symptoms with 50% prevalence). Statistical significance was set at a two-tailed p-value ≤ 0.05.

All statistical analyses were performed using STATA version 15.1 (College Station, USA).

## Results

Of the 1561 patients tested in the two laboratories between March 24th and May 7th 2020, 1543 agreed to participate and were included in the study (participation rate: 99%). They were patients living in and around Greater Lyon. Table 1 summarizes their socio-demographic characteristics and medical conditions. They were predominantly female (63%) and their median age was 44 years (min 1, max 94). More than one-fourth were healthcare professionals (434 patients). The most common medical problems encountered were asthma (13%) and hypertension (11%). Of the 1543 RT-PCR tests performed, 253 were positive for SARS-CoV-2 (16%). The proportion of positive tests decreased over time. In March, the proportion was 28%, in the first two weeks of April 22%, in the last two weeks of April 9%, and in May 4%. As expected, health professionals were younger (median age: 37 years vs. 52) and healthier than other patients (Appendix 1).

**Table 1:** Sociodemographic characteristics and medical conditions of 1543 patients included in the study.

Characteristics	N (%) or median (IQR)
Female gender (n = 1543)	964 (62.5)
Median age (n = 1543)	44 (27)
Health care provider (n = 1225)	434 (35.4)
**Medical conditions (n = 1225)**	
Asthma	154 (12.6)
Hypertension	132 (10.8)
Immunosuppression	73 (6.0)
Diabetes	58 (4.7)
Lung disease^1^	45 (3.7)
Pregnancy	38 (3.1)
Stroke or ischemic heart disease^2^	29 (2.4)
Heart failure	27 (2.2)
Obesity	26 (2.1)
Cancer	16 (1.3)

^1^Number of available data: 1224.

^2^Number of available data: 1223.

Table 2 shows the proportion of symptoms reported by patients who tested negative and positive for SARS-CoV-2. The three most frequently reported ENT and non-ENT symptoms in patients who tested positive were dry throat (42%), loss of smell (36%) and loss of taste (31%), respectively fever (58%), cough (52%) and headache (45%). Compared to patients who tested negative, those who tested positive were significantly more likely to complain of dry nose, loss of taste, loss of smell, fever and muscle pain. On the other hand, they complained significantly less frequently of sore throat, stuffy nose, chest pain, headache, dyspnea and diarrhea. Appendix 2 shows the proportion of symptoms in health professionals and other patients. The results were similar in both patient categories, except for stuffy nose, chest pain and headache. Among health professionals, the proportion of these three symptoms was almost identical in patients who tested negative and positive. In other patients, these symptoms were significantly more often reported by those who tested negative compared to those who tested positive.

**Table 2:** Proportion of symptoms reported by patients with negative and positive SARS-CoV-2 RT-PCR test (N = 1543).

Symptoms	Patients with negative test, N (%)	Patients with positive test, N (%)	p-value^1^
**ENT symptoms**			
Dry throat (n = 1476)	506 (41.3)	106 (42.1)	0.76
Dry nose (n = 1475)	225 (18.4)	67 (26.6)	< 0.001
Sore throat (n = 1517)	97 (7.7)	10 (4.0)	< 0.001
Stuffy nose (n = 1477)	410 (33.5)	59 (23.4)	0.02
Loss of taste (n = 1543)	71 (5.5)	79 (31.2)	< 0.001
Loss of smell (n = 1543)	90 (7.0)	90 (35.6)	< 0.001
Loss of taste and smell (n = 1543)	42 (3.3)	56 (22.1)	< 0.001
Loss of taste or smell (n = 1543)	119 (9.2)	113 (44.7)	< 0.001
**Other symptoms**			
Chest pain (n = 1478)	264 (21.5)	43 (17.1)	< 0.001
Fever (n = 1497)	556 (44.7)	145 (57.5)	0.04
Fatigue (n = 1517)	214 (16.9)	33 (13.2)	0.08
Headache (n = 1487)	653 (52.9)	114 (45.2)	< 0.001
Cough (n = 1517)	563 (44.5)	130 (51.8)	0.27
Muscle pain (n = 1517)	207 (16.4)	64 (25.5)	0.001
Dyspnea (n = 1517)	200 (15.8)	23 (9.2)	< 0.001
Diarrhea (n = 1480)	358 (29.2)	48 (19.1)	0.04

^1^Univariate logistic regression (adjusted for clustering within labs).

Table 3 shows the association between these symptoms and test positivity in unadjusted and adjusted analyses. In multivariable analysis, loss of taste (OR 3.8 [95% CI 3.3–4.4]), loss of smell (OR 3.0 [95% CI 1.9–4.8]), muscle pain (OR 1.6 [95% CI 1.2–2.0]) and dry nose (OR 1.3 [95% CI 1.1–1.6]) were significantly associated with a positive result. The strength of the association with smell and taste disorders was higher for symptom combinations (OR 6.5 [95% CI 3.9–10.8] for loss of taste and smell, OR 6.7 [95% CI 5.9–7.5] for loss of taste or smell). In contrast, sore throat (OR 0.6 [95% CI 0.4–0.8]), stuffy nose (OR 0.6 [95% CI 0.6–0.7]), diarrhea (OR 0.6 [95% CI 0.5–0.6]) and dyspnea (OR 0.5 [95% CI 0.3–0.7]) were inversely associated with a positive test.

**Table 3:** Association between commonly reported symptoms, and positivity of the SARS-CoV-2 RT-PCR test (unadjusted and adjusted analysis).

Symptoms	Crude OR (95% CI)	p-value^1^	Adjusted OR (95%CI)^2^	Adjusted p-value^3^
**ENT symptoms**				
Dry throat (n = 1476)	1.0 (0.9–1.2)	0.76	0.9 (0.6–1.2)	0.41
Dry nose (n = 1475)	1.6 (1.6–1.6)	< 0.001	1.3 (1.1–1.6)	0.01
Sore throat (n = 1517)	0.5 (0.4–0.7)	< 0.001	0.6 (0.4–0.8)	0.003
Stuffy nose (n = 1477)	0.6 (0.4–0.9)	0.02	0.6 (0.6–0.7)	< 0.001
Loss of taste (n = 1543)	7.8 (6.2–9.9)	< 0.001	3.8 (3.3–4.4)	< 0.001
Loss of smell (n = 1543)	7.4 (5.8–9.4)	< 0.001	3.0 (1.9–4.8)	< 0.001
Loss of taste and smell (n = 1543)	8.5 (4.3–16.7)	< 0.001	6.5 (3.9–10.8)	< 0.001
Loss of taste or smell (n = 1543)	8.0 (7.2–8.8)	< 0.001	6.7 (5.9–7.5)	< 0.001
**Other symptoms**				
Chest pain (n = 1478)	0.8 (0.7–0.8)	< 0.001	0.9 (0.7–1.1)	0.32
Fever (n = 1497)	1.7 (1.0–2.8)	0.04	2.1 (0.7–5.9)	0.18
Fatigue (n = 1517)	0.7 (0.5–1.0)	0.08	0.7 (0.4–1.1)	0.11
Headache (n = 1487)	0.7 (0.6–0.9)	< 0.001	1.0 (1.0–1.1)	0.46
Cough (n = 1517)	1.3 (0.8–2.3)	0.27	1.0 (0.7–1.3)	0.81
Muscle pain (n = 1517)	1.8 (1.3–2.5)	0.001	1.6 (1.2–2.0)	0.001
Dyspnea (n = 1517)	0.5 (0.4–0.8)	< 0.001	0.5 (0.3–0.7)	< 0.001
Diarrhea (n = 1480)	0.6 (0.3–1.0)	0.04	0.6 (0.5–0.6)	< 0.001

^1^Univariate logistic regression (adjusted for clustering within labs).

^2^Number of available data: 1194.

^3^Multivariable logistic regression (adjusted for clustering within labs, gender, age group, patient population (health care provider vs. other), RT-PCR date (March, April or May), and all symptoms listed in the table).

Table 4 shows the diagnostic performance for all symptoms. The two symptoms with the highest diagnostic performance were loss of taste (AUC 0.63) and loss of smell (AUC 0.64). The combination of the two symptoms (loss of taste or smell), despite a slight decrease in specificity, resulted in improved diagnostic performance (AUC 0.68) due to increased sensitivity. In addition, positive predictive values ranged from 9% for sore throat to 53% for loss of taste, while negative predictive values ranged from 81% for stuffy nose, headache and diarrhea to 88% for loss of taste and loss of smell. The two symptoms with the highest positive and negative predictive values were again loss of taste and loss of smell. These values were 53% and 88% for loss of taste, 50% and 88% for loss of smell, respectively. Combining the two symptoms, these figures were slightly modified (57% and 86% in the presence of both symptoms, and 49% and 89% in the presence of at least one of the two symptoms). Combination with other symptoms did not improve the diagnostic performance any further.

**Table 4:** Diagnostic performance of commonly reported symptoms.

Symptom	Sensitivity, % (95%CI)	Specificity, % (95%CI)	ROC area (95%CI)	Positive predictive value, % (95%CI)	Negative predictive value, % (95%CI)
**ENT symptoms**					
Dry throat (n = 1476)	42.1 (35.9–48.4)	58.7 (55.8–61.4)	0.50 (0.47–0.54)	17.3 (14.4–20.6)	83.1 (80.4–85.5)
Dry nose (n = 1475)	26.6 (21.2–32.5)	81.6 (79.3–83.7)	0.54 (0.51–0.57)	22.9 (18.2–28.2)	84.4 (82.2–86.4)
Sore throat (n = 1517)	4.0 (1.9–7.2)	92.3 (90.7–93.7)	0.48 (0.47–0.50)	9.4 (4.6–16.5)	82.9 (80.8–84.8)
Stuffy nose (n = 1477)	23.4 (18.3–29.1)	66.5 (63.8–69.2)	0.45 (0.42–0.48)	12.6 (9.7–15.9)	80.9 (78.3–83.2)
Loss of taste (n = 1543)	31.2 (25.6–37.3)	94.5 (93.1–95.7)	0.63 (0.60–0.66)	52.7 (44.4–60.9)	87.5 (85.7–89.2)
Loss of smell (n = 1543)	35.6 (29.7–41.8)	93.0 (91.5–94.4)	0.64 (0.61–0.67)	50.0 (42.5–57.5)	88.0 (86.2–89.7)
Loss of taste and smell (n = 1543)	22.1 (17.2–27.8)	96.7 (95.6–97.6)	0.59 (0.57–0.62)	57.1 (46.7–67.1)	86.4 (84.5–88.1)
Loss of taste or smell (n = 1543)	44.7 (38.4–51.0)	90.8 (89.1–92.3)	0.68 (0.65–0.71)	48.7 (42.1–55.3)	89.3 (87.5–90.9)
**Other symptoms**					
Chest pain (n = 1478)	17.1 (12.6–22.3)	78.5 (76.1–80.7)	0.48 (0.45–0.50)	14.0 (10.3–18.4)	82.2 (79.8–84.3)
Fever (n = 1497)	57.5 (51.2–63.7)	55.3 (52.5–58.1)	0.56 (0.53–0.60)	20.7 (17.7–23.9)	86.6 (84.0–88.9)
Fatigue (n = 1517)	13.1 (9.2–18.0)	83.1 (80.9–85.1)	0.48 (0.46–0.51)	13.4 (9.4–18.2)	82.8 (80.6–84.9)
Headache (n = 1487)	45.2 (39.0–51.6)	47.1 (44.3–50.0)	0.46 (0.43–0.50)	14.9 (12.4–17.6)	80.8 (77,8–83.6)
Cough (n = 1517)	51.8 (45.4–58.1)	55.5 (52.7–58.3)	0.54 (0.50–0.57)	18.8 (15.9–21.9)	85.3 (82.7–87.7)
Muscle pain (n = 1517)	25.5 (20.2–31.4)	83.6 (81.5–85.6)	0.55 (0.52–0.58)	23.6 (18.7–29.1)	85.0 (82.9–86.9)
Dyspnea (n = 1517)	9.2 (5.9–13.4)	84.2 (82.1–86.2)	0.47 (0.45–0.49)	10.3 (6.7–15.1)	82.4 (80.2–84.4)
Diarrhea (n = 1480)	19.0 (14.4–24.4)	70.8 (68.2–73.4)	0.45 (0.42–0.48)	11.8 (8.9–15.4)	81.0 (78.5–83.3)

We repeated the multivariable analysis after removing “patient population” from the variables included in the analysis (Appendix 3). Indeed, this variable alone had 318 missing data (vs. less than 100 for the other variables). The strengths of association obtained in both multivariable analyses (with and without "patient population") were similar.

Serological data were available two months after the initial data collection for 70 patients with a negative RT-PCR, and were positive in 3 cases.

## Discussion

### Summary

Our study sample consisted of 1543 primary care patients tested in two laboratories in the Lyon area (France), with 16% positive tests for SARS-CoV-2. We found that dry nose, loss of taste and/or smell and muscle pain were more frequent in patients with a positive test, while sore throat, stuffy nose, dyspnea and diarrhea were more frequent in patients with a negative result. We also found that the two symptoms most strongly associated with a positive test were loss of taste and smell, and that the combination of these two symptoms resulted in an even stronger association.

### Comparison with existing literature

Many researchers were interested in describing the clinical presentations of COVID-19 patients. Although the majority of studies involved hospital or outpatient clinic populations, some studies were also carried out in the general population or in primary care^7–9,18–20,26^. The results obtained are relatively variable. Considering only community samples, whether in primary care or not, two symptoms were consistently found more frequently in SARS-CoV-2 patients compared to others, namely taste and smell disorders. In contrast, all other symptoms were associated in some studies but not in others with SARS-CoV-2 infection. This was also true for symptoms examined in all studies, such as cough or fever. For example, cough was more common in SARS-CoV-2 patients compared to uninfected individuals in an Italian web-based survey (EPICOVID19)^7,8^, but not in ours or in other studies conducted with community samples^9,16,17^. This variability in results probably reflects important differences in study design, including sample size, population examined (patients vs general population) and type of recruitment.

The data from our study also differed, to a greater extend, from those from studies conducted in China, which mainly involved hospitalized patients with severe infections^10,24,27–29^. For example, in the study by Huang et al., the most frequently reported symptoms were fever (98%), cough (76%) and dyspnea (55%)^29^, while in the study by Wang et al., the most prevalent symptoms were fever (99%), fatigue (70%) and cough (59%)^24^.

As already suggested in our preliminary study^19^, we confirm that the two symptoms most strongly associated with a positive test were loss of taste and loss of smell. Interestingly, the combination of these two symptoms results in an even stronger association, and also has the best diagnostic performance. Several other authors have recently published similar results, particularly on European outpatient populations^7–9,16,17,22,26,30^. SARS-CoV-2 has been shown to have a particular tropism for the nerves of the ear, nose and throat system^31^. This is probably the reason why the proportion of patients with taste and smell disorders is higher in COVID-19 patients than in patients with other respiratory tract infections. The fact that in our study COVID-19 patients complained less frequently of stuffy nose than other patients (23% vs. 34%) is consistent with this hypothesis.

We found that patients who tested positive were less likely to complain of dyspnea and diarrhea than patients who tested negative. This result is rather counter-intuitive as several authors showed that these two symptoms were relatively common in patients infected with SARS-CoV-2^10,16,24,27,28^. However, these were mainly data from hospital-based studies. The clinical pictures presented by these patients are not necessarily similar to those presented by outpatients with mild to moderate symptoms. For example, patients often require hospitalization because of oxygen desaturation due to lung involvement. Its high prevalence among inpatients certainly explains why they have more dyspnea than our community-based sample.

### Implications for research and/or practice

Taste and smell disorders were the most specific symptoms of SARS-CoV-2 infection in our sample, and the specificity increased further when these two symptoms were combined (loss of taste and smell). However, the diagnostic performance of combined loss of taste and smell was not sufficient to confirm infection with sufficient safety in affected patients. Indeed, the positive predictive value (i.e. the probability of testing positive) was only 57% in the presence of both symptoms (56 out of 98 patients). Furthermore, this diagnostic performance is expected to decrease as the prevalence of infection decreases. In addition, despite higher numbers, negative predictive values did not rule out infection in patients without taste and/or smell disorders. The negative predictive value was identical (88%) for loss of taste (174 out of 1393 patients were positive despite the absence of this symptom) and for loss of smell (163 out of 1363 patients). The combination of the two symptoms (loss of taste or smell) resulted in a slight increase in the negative predictive value (89%) but 140 patients were still misdiagnosed as uninfected among 1311 symptomatic patients.

The gold standard for our study was a PCR test. This test is also imperfect and a false negative result (i.e. a negative PCR test in an infected patient) cannot be ruled out. At the time of the study, serological tests were not yet readily available in France. However, we were able to retrieve results from 70 patients with a negative PCR test. Among these patients, serology was positive in only 3 patients and negative in the other 67. Although serology testing could only be performed in a small number of patients, this 4% false negative result makes us believe that our gold standard was appropriate for the needs of the study. Nevertheless, it would be useful to conduct further studies in primary care combining both tests (PCR and serology).

### Strengths and limitations

The study took place in a single French region (Greater Lyon). Our results are therefore not necessarily generalizable to other regions in France or to other countries. Due to the heavy workload in the SARS-CoV-2 screening laboratories, we did not ask the healthcare professionals receiving the patients to report the characteristics of those who refused to participate. We do not know if these 18 patients had different clinical presentations than those who agreed to participate. As already stated, our results are also limited by the diagnostic performance of the nasal swabs for RT-PCR testing. Serological tests for SARS-CoV-2 were not done systematically since they were not widely available at the time of the study. The diagnostic performance of the symptoms could have been higher if, for example, RT-PCR tests had been combined with serological tests for all patients in our study. Finally, our study only included patients who were stable enough to undergo RT-PCR testing in a community setting. As such they may not represent the entire spectrum of patients presenting to primary care, since more severely affected patients with a suspicion of SARS-CoV-2 are likely to have been referred directly to the hospital. However, since telephone triage processes were in place in all practices at the time of the study, most patients with severe respiratory symptoms are likely to have been referred directly to the hospital, without a prior visit to the GP.

## Conclusion

In conclusion, we found that dry nose, loss of taste and/or smell and muscle pain were more frequent in patients with a positive test, while sore throat, stuffy nose, dyspnea and diarrhea were more frequent in patients with a negative result. We confirmed that the two symptoms most strongly associated with a positive test were loss of taste and loss of smell, and that the combination of these two symptoms resulted in an even stronger association. These results could contribute to the triage and early identification of new clusters of cases.

## Supplementary Information

Supplementary Appendix 1.

Supplementary Appendix 2.

Supplementary Appendix 3.
