# Supplementary material for: Cross sectional study of the clinical characteristics of French primary care patients with COVID-19
Source: Sci Rep. 2021 Jun 14;11:12492. doi: 10.1038/s41598-021-91685-3 (PMC8203628; doi:10.1038/s41598-021-91685-3)
Supplement: Supplementary file 1 — Supplementary Appendix 1. [file 41598_2021_91685_MOESM1_ESM.docx]

**Cross sectional study of the clinical characteristics of French primary care patients with COVID-19**

Paul Sebo, Benoit Tudrej, Julie Lourdaux, Clara Cuzin, Martin Floquet, Dagmar M. Haller, Hubert Maisonneuve

Appendix #1. Sociodemographic characteristics and medical conditions, stratified by study population (healthcare professionals vs. other patients)

| Characteristics | Healthcare professionals (n=434), N (%) | Other patients (n=791), N (%) | p-value^1^ |
| --- | --- | --- | --- |
| Female gender (n=1225) | 346 (79.7) | 437 (55.3) | <0.001 |
| Age group (years) (n=1225) |  |  | <0.001 |
| < 40 | 240 (55.3) | 239 (30.2) |  |
| 40-59 | 162 (37.3) | 262 (33.1) |  |
| ≥ 60 | 32 (7.4) | 290 (36.7) |  |
| Medical conditions (n=1225) |  |  |  |
| Asthma | 7 (1.6) | 147 (18.6) | <0.001 |
| Hypertension | 8 (1.8) | 124 (15.7) | <0.001 |
| Immunosuppression | 2 (0.5) | 71 (9.0) | 0.002 |
| Diabetes | 1 (0.2) | 57 (7.2) | <0.001 |
| Lung disease^2^ | 1 (0.2) | 44 (5.6) | <0.001 |
| Pregnancy | 1 (0.2) | 37 (4.7) | <0.001 |
| Stroke or ischemic heart disease^3^ | 0 | 29 (3.7) | <0.001 |
| Heart failure | 1 (0.2) | 26 (3.3) | 0.01 |
| Obesity^2^ | 0 | 26 (3.3) | <0.001 |
| Cancer | 0 | 16 (2.0) | <0.001 |

^1^ univariate logistic regression (adjusted for clustering within labs)

^2^ number of available data: 1224

^3^ number of available data: 1223
