# Supplementary material for: Cross sectional study of the clinical characteristics of French primary care patients with COVID-19
Source: Sci Rep. 2021 Jun 14;11:12492. doi: 10.1038/s41598-021-91685-3 (PMC8203628; doi:10.1038/s41598-021-91685-3)
Supplement: Supplementary file 2 — Supplementary Appendix 2. [file 41598_2021_91685_MOESM2_ESM.docx]

**Cross sectional study of the clinical characteristics of French primary care patients with COVID-19**

Paul Sebo, Benoit Tudrej, Julie Lourdaux, Clara Cuzin, Martin Floquet, Dagmar M. Haller, Hubert Maisonneuve

Appendix #2. Proportion of symptoms reported by participants with negative and positive SARS-CoV-2 RT-PCR test, stratified by study population (healthcare professionals vs. other patients)

| Symptoms | Healthcare professionals with negative test (N=337), N (%) | Healthcare professionals with positive test (N=97), N (%) | p-value^1^ | Other patients with negative test (N=655), N (%) | Other patients with positive test (N=136), N (%) | p-value^1^ |
| --- | --- | --- | --- | --- | --- | --- |
| ENT symptoms |  |  |  |  |  |  |
| Dry throat | 137 (41.5) | 41 (42.7) | 0.76 | 273 (43.1) | 56 (41.2) | 0.62 |
| Dry nose | 70 (21.3) | 28 (29.2) | 0.01 | 118 (18.6) | 34 (25.0) | <0.001 |
| Sore throat | 26 (7.7) | 5 (5.2) | <0.001 | 47 (7.2) | 4 (2.9) | <0.001 |
| Stuffy nose | 102 (31.0) | 31 (32.3) | 0.84 | 213 (33.5) | 21 (15.4) | <0.001 |
| Loss of taste | 26 (7.7) | 37 (38.1) | <0.001 | 42 (6.4) | 40 (29.4) | <0.001 |
| Loss of smell | 35 (10.4) | 37 (38.1) | <0.001 | 51 (7.8) | 50 (36.8) | <0.001 |
| Loss of taste and smell | 15 (4.5) | 26 (26.8) | <0.001 | 25 (3.8) | 28 (20.6) | <0.001 |
| Loss of taste or smell | 46 (13.7) | 48 (49.5) | <0.001 | 68 (10.4) | 62 (45.6) | <0.001 |
| Other symptoms |  |  |  |  |  |  |
| Chest pain | 55 (16.7) | 16 (16.7) | 0.91 | 151 (23.8) | 23 (16.9) | <0.001 |
| Fever | 133 (40.2) | 45 (46.9) | <0.001 | 279 (43.5) | 89 (65.4) | 0.12 |
| Fatigue | 66 (19.6) | 14 (14.4) | <0.001 | 100 (15.3) | 17 (12.5) | 0.52 |
| Headache | 177 (53.6) | 52 (54.2) | 0.05 | 312 (49.0) | 50 (36.8) | <0.001 |
| Cough | 146 (43.5) | 45 (46.4) | 0.77 | 320 (48.9) | 73 (53.7) | <0.001 |
| Muscle pain | 67 (19.9) | 32 (33.0) | <0.001 | 100 (15.3) | 29 (21.3) | 0.001 |
| Dyspnea | 49 (14.6) | 10 (10.3) | <0.001 | 133 (20.3) | 13 (9.6) | 0.004 |
| Diarrhea | 85 (25.8) | 15 (15.6) | 0.26 | 172 (27.1) | 26 (19.1) | <0.001 |

^1^ univariate logistic regression (adjusted for clustering within labs)
