# Supplementary material for: Cross sectional study of the clinical characteristics of French primary care patients with COVID-19
Source: Sci Rep. 2021 Jun 14;11:12492. doi: 10.1038/s41598-021-91685-3 (PMC8203628; doi:10.1038/s41598-021-91685-3)
Supplement: Supplementary file 3 — Supplementary Appendix 3. [file 41598_2021_91685_MOESM3_ESM.docx]

**Cross sectional study of the clinical characteristics of French primary care patients with COVID-19**

Paul Sebo, Benoit Tudrej, Julie Lourdaux, Clara Cuzin, Martin Floquet, Dagmar M. Haller, Hubert Maisonneuve

Appendix #3. Association between commonly reported symptoms, and positivity of the SARS-CoV-2 RT-PCR test (unadjusted and adjusted analysis)

| Symptoms | Crude OR (95% CI) | p-value^1^ | Adjusted OR (95%CI)^2^ | Adjusted p-value^3^ |
| --- | --- | --- | --- | --- |
| ENT symptoms |  |  |  |  |
| Dry throat | 1.0 (0.9-1.2) | 0.76 | 0.9 (0.7-1.1) | 0.29 |
| Dry nose | 1.6 (1.6-1.6) | <0.001 | 1.4 (1.3-1.4) | <0.001 |
| Sore throat | 0.5 (0.4-0.7) | <0.001 | 0.6 (0.3-1.0) | 0.06 |
| Stuffy nose | 0.6 (0.4-0.9) | 0.02 | 0.7 (0.6-0.8) | <0.001 |
| Loss of taste | 7.8 (6.2-9.9) | <0.001 | 3.7 (3.6-3.7) | <0.001 |
| Loss of smell | 7.4 (5.8-9.4) | <0.001 | 3.0 (1.9-4.8) | <0.001 |
| Loss of taste and smell | 8.5 (4.3-16.7) | <0.001 | 6.3 (3.4-11.9) | <0.001 |
| Loss of taste or smell | 8.0 (7.2-8.8) | <0.001 | 6.4 (6.4-6.4) | <0.001 |
| Other symptoms |  |  |  |  |
| Chest pain | 0.8 (0.7-0.8) | <0.001 | 0.9 (0.7-1.0) | 0.06 |
| Fever | 1.7 (1.0-2.8) | 0.04 | 1.8 (0.7-4.5) | 0.20 |
| Fatigue | 0.7 (0.5-1.0) | 0.08 | 0.7 (0.6-0.9) | 0.01 |
| Headache | 0.7 (0.6-0.9) | <0.001 | 1.0 (0.9-1.1) | 0.89 |
| Cough | 1.3 (0.8-2.3) | 0.27 | 1.1 (0.6-1.8) | 0.81 |
| Muscle pain | 1.8 (1.3-2.5) | 0.001 | 1.5 (0.9-2.5) | 0.12 |
| Dyspnea | 0.5 (0.4-0.8) | <0.001 | 0.5 (0.3-0.7) | <0.001 |
| Diarrhea | 0.6 (0.3-1.0) | 0.04 | 0.6 (0.5-0.7) | <0.001 |

^1^ univariate logistic regression (adjusted for clustering within labs)

^2^ number of available data: 1449

^3^ multivariable logistic regression (adjusted for clustering within labs, gender, age group, RT-PCR date (March, April or May), and all symptoms listed in the table, but without adjusting for health professionals versus other patients)
